# Foot posture in people with medial compartment knee osteoarthritis

**DOI:** 10.1186/1757-1146-3-29

**Published:** 2010-12-16

**Authors:** Pazit Levinger, Hylton B Menz, Mohammad R Fotoohabadi, Julian A Feller, John R Bartlett, Neil R Bergman

**Affiliations:** 1 Musculoskeletal Research Centre, Faculty of Health Sciences, La Trobe University. Bundoora, Victoria 3086, Australia; 2 Warringal Medical Centre, Heidelberg, Victoria 3084, Australia

## Abstract

**Background:**

Foot posture has long been considered to contribute to the development of lower limb musculoskeletal conditions as it may alter the mechanical alignment and dynamic function of the lower limb. This study compared foot posture in people with and without medial compartment knee osteoarthritis (OA) using a range of clinical foot measures. The reliability of the foot measures was also assessed.

**Methods:**

The foot posture of 32 patients with clinically and radiographically-confirmed OA predominantly in the medial compartment of the knee and 28 asymptomatic age-matched healthy controls was investigated using the foot posture index (FPI), vertical navicular height and drop, and the arch index. Independent t tests and effect size (Cohen's d) were used to investigate the differences between the groups in the foot posture measurements.

**Results:**

Significant differences were found between the control and the knee OA groups in relation to the FPI (1.35 ± 1.43 vs. 2.46 ± 2.18, p = 0.02; *d *= 0.61, medium effect size), navicular drop (0.02 ± 0.01 vs. 0.03 ± 0.01, p = 0.01; *d *= 1.02, large effect size) and the arch index (0.22 ± 0.04 vs. 0.26 ± 0.04, p = 0.04; *d *= 1.02, large effect size). No significant difference was found for vertical navicular height (0.24 ± 0.03 vs. 0.23 ± 0.03, p = 0.54; *d *= 0.04, negligible effect size).

**Conclusion:**

People with medial compartment knee OA exhibit a more pronated foot type compared to controls. It is therefore recommended that the assessment of patients with knee OA in clinical practice should include simple foot measures, and that the potential influence of foot structure and function on the efficacy of foot orthoses in the management of medial compartment knee OA be further investigated.

## Background

Knee osteoarthritis (OA) is a common painful and chronic condition that affects a large proportion of the older population [1,2]. Knee OA may in part be due to excessive loading of the articular cartilage [3]. During walking, the forces transmitted across the knee joint are greater in the medial compartment compared to the lateral compartment [4], and increased medial compartment loading has been observed in patients with knee OA [5-8]. The mechanics of gait, in particular the knee adduction moment (the moment that tends to adduct the knee during the stance phase of walking), have been shown to be a contributing factor to the progression of medial compartment knee OA [5-7,9]. Treatment strategies for knee OA, such us foot orthoses, knee braces and footwear, have been proposed to minimise the knee adduction moment, and consequently reduce the loading on the medial compartment [10-18].

Foot posture has long been considered to contribute to the development of a range of lower limb musculoskeletal conditions [19,20] as it may alter the mechanical alignment and dynamic function of the lower limb [21]. Special attention, therefore, has been given to foot orthoses and footwear modifications as a non-operative treatment of knee OA [13,15,18,22,23]. However, in order to fully understand the effect of these interventions on the knee and other lower limb joints and to identify patients who are most likely to benefit from them, greater knowledge of foot structure in this population is required.

Despite the potential importance of understanding foot characteristics of people with medial compartment knee OA, few studies have examined foot posture in this population. Reilly et al [24] compared navicular height in sitting and standing in 60 people with hip OA, 60 people with knee OA and 60 controls, and found no differences between the knee OA and control groups. However, there was a significant difference in frontal plane calcaneal angle, indicating a more everted rearfoot in the knee OA group. In a subsequent study, these authors also compared foot posture index (FPI) scores between 20 people with knee OA and 20 controls, and reported a significantly higher median score in those with knee OA (7.0 versus 1.0), indicative of a more pronated foot posture [25].

A key consideration when interpreting these findings is the reliability of the foot posture measures. Previous studies have indicated that frontal plane calcaneal measures have questionable reliability [26], while FPI reliability is moderate to good, depending on the clinical experience of the assessor [27]. Given the questionable reliability reported for some of the foot measures and the expertise required to take these measures [26-30], using an objective measure that does not require any subjective interpretation may be important to include as part of foot posture assessment. However, evaluation of such a measure in people with knee OA has not previously been investigated. The primary aim of this study therefore was to investigate foot type in people with and without medial compartment knee OA using a range of clinical foot measures, including a measure (the arch index) that requires no clinical expertise or subjective interpretation. A secondary aim was to determine the reliability of the foot posture measurements.

## Methods

Two groups participated in the study: a knee OA group and an age-matched asymptomatic control group. The OA group included 32 participants diagnosed with predominantly medial compartment OA, determined by radiographic assessment. The severity of knee OA was based on the loss of joint space determined by an orthopaedic surgeon from radiographic images [31] and was graded as follows: 1- less than a half of joint space loss (mild), 2 - more than a half of joint space loss; bone on bone (moderate) and 3 - bone deformity/loss of bone (severe). Each compartment of the knee joint (medial compartment, lateral compartment and patellofemoral compartment) was graded and participants with predominantly medial compartment OA (severity grade 2-3) were included in the study. Participants from the OA group were included if they were able to walk independently and were excluded if they had uncontrolled systemic disease and or a pre-existing neurological or other orthopaedic condition that affected their walking. Participants from the OA group were recruited from the La Trobe University Medical Centre, the Warringal Private Medical Centre and through advertisements in local newspapers. The control group consisted of 28 asymptomatic participants with no clinical diagnosis of OA, rheumatoid arthritis or history of knee trauma or pain. Participants from the control group were recruited from retirement villages in northern Melbourne and through advertisements in local newspapers. Ethics approval was obtained from the Faculty of Health Sciences Human Ethics Committee, La Trobe University. All participants were informed about the nature of the study and signed a consent form prior to participation.

### Procedure

All participants attended the gait laboratory at La Trobe University for a single session, and 23 participants from the control group attended on two occasions to assess the reliability of the foot measurements. All foot measurements were assessed by the same examiner (PL) with previous experience in taking these measures [27]. Participants' body mass, height and truncated foot length were recorded. The symptomatic leg (or the most symptomatic leg in a case of bilateral involvement) in the OA group and the same corresponding leg of each peer control matched for age were assessed.

### Foot posture measurements

The foot posture measurements included the foot posture index (FPI), navicular height, navicular drop and the arch index. The FPI is a 6-item foot posture assessment with the subject standing relaxed in a bipedal position [29]. The 6 items of the FPI include talar head palpation, curves above and below the lateral malleoli, calcaneal angle, talonavicular bulge, medial longitudinal arch and forefoot to rearfoot alignment. Each item was scored on a 5-point scale between -2 and +2 and provides a total sum of all items between -12 (highly supinated) and +12 (highly pronated). The raw FPI scores were converted to Rash transformed scores to allow the scores to be used as interval data [32]. The transformed FPI values were used for the analysis.

**Figure 1:**
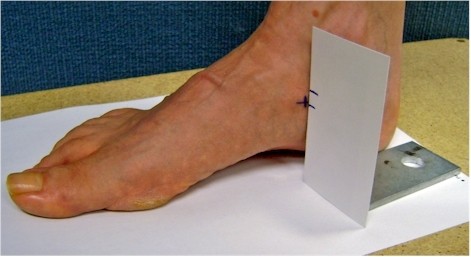
**Navicular height and drop measurement**.

Navicular height and navicular drop measurements were taken in subtalar joint neutral (STJN) position and in relaxed standing posture using a business card as described previously [33] and with the aid of a right-angled metal bracket for stabilising the card [27]. STJN was defined as the position of the foot when the talar head could be palpated just anterior to the ankle mortise with equal prominence both medially and laterally. The position of the subtalar joint in neutral was maintained and the vertical height of the navicular was marked on the business card. The participants were then asked to relax and the vertical height of the navicular was marked on the card. Navicular drop was measured as the difference between the STJN and relaxed stance of the navicular height (see Figure 1). Both measures were normalised to each participant's truncated foot length. Truncated foot length was measured from the most posterior aspect of the calcaneus to the first metatarsophalangeal joint. Truncated foot length was used for normalisation due to the potential presence of toe deformity in older people which can affect the foot length value [34].

**Figure 2:**
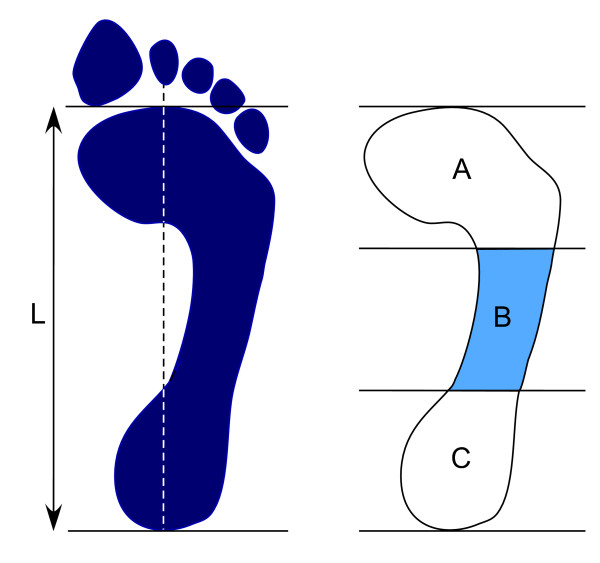
**Calculation of the AI**. The truncated length of the footprint (L) is divided into equal thirds. The AI is then calculated as the area of the middle third of the footprint divided by the entire footprint area (AI = B/[A + B + C]).

The arch index was measured with the participant standing on a carbon paper imprint material in relaxed bipedal stance. A static footprint was obtained and was divided to three equal sections. The arch index was then calculated as the ratio of the middle section to the entire footprint area using a computer graphics tablet (Wacom Technology Corporation, Vancouver, Canada). Higher values of the arch index indicate a flatter (more pronated) foot [35]. See Figure 2.

### Statistical analysis

All analyses were performed using SPSS 17.0 for Windows (SPSS Inc., Chicago IL, USA). The intra-rater reliability of the foot posture measurements was evaluated using intraclass correlation coefficients (ICCs_3,1_), 95% limits of agreement and coefficient of variation [36]. ICCs above 0.90 were considered excellent, 0.75 - 0.90 considered good, 0.50 - 0.75 considered moderate and ICC below 0.50 considered poor [37]. Differences between the groups were assessed using independent samples t-tests for continuously scored variables and chi-squared statistics for categorical variables. The magnitude of the differences in continuously-scored variables between the groups was assessed using Cohen's *d*, with the following cut-offs applied to aid interpretation: <0.15 - negligible effect, ≥ 0.15 to <0.40 - small effect, ≥ 0.40 to <0.75 - medium effect, ≥ 0.75 to <1.10 - large effect, ≥ 1.10 to <1.45 - very large effect, and >1.45 - huge effect [38]. To explore the potential correlation between body weight and the foot posture measures, Pearson's correlation coefficient was used. Where significant correlations were found, bodyweight was used as a covariate for that particular foot posture measure.

## Results

The demographic characteristics of both groups are summarised in Table 1. The participants' age and height were similar between the groups, although the knee OA group had a significantly greater body weight and body mass index. The ICCs for the foot measures ranged from moderate to excellent. Navicular height and drop showed ICC = 0.86 and ICC = 0.56, respectively, with FPI and arch index having ICC = 0.91 and ICC = 0.93, respectively. Similarly, low coefficients of variation were found for the FPI, navicular height and arch index (Table 2).

**Table 1:** Participants' demographic characteristics

Parameters	Control group (n = 28)	Knee OA group (n = 32)	p value
Age - yr	65.22 ± 11.41	65.84 ± 7.57	0.810
Female - n (%)	15 (54)	16 (46)	0.210
Height - cm	168.61 ± 10.64	168.83 ± 9.54	0.932
Body weight - kg	73.12 ± 15.49	85.13 ± 13.67	0.003*
Body mass index - kg/m^2^	25.56 ± 3.95	29.97 ± 5.26	0.001*

Values are reported as mean ± SD unless otherwise noted.* significant at p < 0.05.

**Table 2:** Reliability of the foot posture measurements.

Measures	Session 1 mean ± SD	Session 2 mean ± SD	**ICC**_**3,1 **_**(95% CI)**	95% LoA	CV (%)
Foot posture index†	1.33 ± 1.47	1.46 ± 1.33	0.91 (0.82 to 0.96)	1.44 to -1.88	24
Navicular height	0.24 ± 0.03	0.23 ± 0.03	0.86 (0.71 to 0.94)	0.04 to -0.03	6
Navicular drop	0.01 ± 0.01	0.01 ± 0.01	0.56 (0.20 to 0.79)	0.02 to -0.02	38
Arch index	0.21 ± 0.04	0.21 ± 0.04	0.93 (0.84 to 0.97)	0.03 to -0.03	5

NB: ICC - intraclass correlation coefficient; LoA - 95% limit of agreement; CV - coefficient of variation. † Rasch transformed FPI scores

A significant correlation was found between body weight and the arch index (*r *= 0.44, *p *< 0.001) with no significant correlation between body weight and FPI (*r *= 0.22, *p *= 0.09), navicular height (*r *= 0.008, *p *= 0.94) or navicular drop (*r *= 0.20, *p *= 0.12). Body weight was therefore entered as a covariate for the comparison of the arch index between the groups.

**Figure 3:**
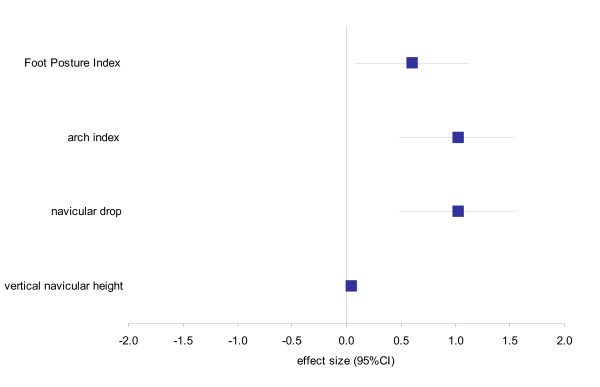
**Effect sizes and 95% confidence intervals for the difference in foot posture variables between the control and knee OA groups**. Positive values indicate larger scores in the knee OA group, negative values indicate larger scores in the control group.

Significant differences were found between the groups for three foot measures, with the knee OA group exhibiting a more pronated foot compared to the control group for the FPI (2.46 ± 2.18 vs 1.35 ± 1.43.; *p *= 0.02; *d *= 0.61, medium effect size), navicular drop (0.03 ± 0.01 vs 0.02 ± 0.01; *p *= 0.01; *d *= 1.02, large effect size) and arch index (0.26 ± 0.04 vs 0.22 ± 0.04; *p *= 0.04; *d *= 1.02, large effect size) as indicated in Figure 3. No significant difference was found between the groups for navicular height (Table 3).

**Table 3:** Differences in foot posture measurements between the groups.

Measure	Control (n = 28)	Knee OA (n = 32)	p value	Effect size (Cohen's d)
Foot posture index†	1.35 ± 1.43	2.46 ± 2.18	0.022*	d = 0.61 (medium)
Navicular height	0.24 ± 0.03	0.23 ± 0.03	0.542	d = 0.04 (negligible)
Navicular drop	0.02 ± 0.01	0.03 ± 0.01	0.019*	d = 1.02 (large)
Arch index	0.22 ± 0.04	0.26 ± 0.04	0.040*	d = 1.02 (large)

Values are reported as mean ± SD.* significant at p < 0.05. † Rasch transformed FPI scores

## Discussion

Foot posture has long been considered to influence the mechanical alignment and dynamic function of the lower limb and may therefore be related to the development of lower limb musculoskeletal conditions. Subsequently, several recent studies have drawn attention to the potential benefits of foot orthoses in reducing the load on the knee, particularly the knee adduction moment [13,15,18,22,23]. Assessing foot characteristics of people with medial compartment OA may therefore advance our understanding of the potential role of foot orthoses and footwear modifications on lower limb alignment and function.

In this study, we investigated foot characteristics of people with medial compartment knee OA using several foot measures. The OA group exhibited a more pronated foot type compared to the control group, as indicated by the three foot measures: FPI, navicular drop and arch index, with medium to large effect sizes. Similar findings were reported by Reilly and colleagues for people with severe knee medial compartment OA using several foot measures, including the FPI [24,25]. However, we found no significant difference in navicular height between the groups, which is also in agreement with Reilly and colleagues [24].

Whether pronated foot posture is a risk factor for, or a consequence of, medial compartment knee OA cannot be determined from cross-sectional studies such as ours. People with medial compartment knee OA often display genu varum malalignment of the knee, which has been shown to increase the risk of development and progression of knee OA [39,40]. Genu varum malalignment of the knee may lead to compensatory foot pronation to enable the foot to be plantigrade when weightbearing [41]. In a recent study, a simulated genu varum walking pattern was found to increase the subtalar joint pronation moment, suggesting that frontal plane angular deformities of the knee can alter the kinetic and kinematics of the foot during gait [42]. Increased foot pronation could potentially reduce the adduction moment by shifting the centre of pressure laterally, so it is possible that the foot adapts to reduce the load on the medial compartment. However, the degree of genu varum that can be compensated by foot pronation depends on the available range of motion of the ankle, subtalar and midtarsal joints [43]. Due to the potential effect of foot alignment on the loading axis of the lower limb, a longitudinal investigation is required to better understand the contribution of foot structure and function to the development of medial compartment knee OA.

The findings reported here may have implications for orthotic and footwear interventions that are commonly suggested for the management of knee OA. In particular, laterally wedged insoles have been proposed for people with medial compartment knee OA, as they have been shown to reduce the knee adduction moment and reduce symptoms [12,13,18,22,23]. However, laterally wedged insoles can alter foot motion, specifically increasing rearfoot pronation [44,45]. Accentuation of rearfoot pronation in already pronated feet could potentially result in detrimental changes to lower limb kinematics, and consequently lead to the development of musculoskeletal problems in other regions. Interestingly, studies have shown that the biomechanical effects of laterally wedged insoles are inconsistent, with some participants exhibiting *increases *in the knee adduction moment [46,47]. Furthermore, Nakajima et al [14] have recently reported that the addition of an arch support to laterally wedged insoles maintains normal rearfoot motion while also enhancing the ability of the insole to reduce the knee adduction moment. These findings indicate that the biomechanical effects of laterally wedged insoles may be influenced by individual variation in foot function. As such, there may be a need to include foot posture screening to appropriately identify those who are most likely to benefit from laterally wedged insoles, in order to guide the selection of modifications such as the addition of arch supports.

The reliability of foot measures has been widely reported in a range of populations [26-30]. In the present study, good to excellent intrarater reliability was found for the navicular height, arch index and FPI which was comparable to previous studies assessing intrarater reliability [27,48,49] where the examiners had experience in taking foot measures. In contrast, the reliability of navicular height was only moderate, which was similar to the reliability reported by Evans et al for an adult population [49]. Measuring navicular drop involves placing the subtalar joint in neutral which requires clinical experience in order to achieve an acceptable level of reliability. However, the examiner in our study had previous experience in taking foot measures with good intrarater and interrater reliability, as we have previously reported in a younger population [27]. We therefore believe that the moderate reliability may be related to the age of our sample. Placing the subtalar joint in neutral during standing may be less reliable in older people as it requires active involvement of the participant [50] which can be challenging due to difficulty in maintaining balance.

The arch index is a reliable tool that quantifies foot characteristics based on a static footprint, and as such does not rely on the clinical experience of the examiner. The arch index however, has not been assessed previously in people with knee OA. Our results indicate that the arch index demonstrates excellent reliability, and can detect differences in foot posture between people with and without medial compartment knee OA. Importantly, the differences between the groups persisted after adjusting for bodyweight, which addresses previous concerns that the arch index may be a measure of 'fat' rather than 'flat' feet [51]. These findings suggest that the arch index may have some clinical utility in the assessment of patients with knee OA.

## Conclusion

People with medial compartment knee OA exhibit a more pronated foot type compared to controls, as indicated by the FPI, navicular drop and arch index. It is therefore recommended that the assessment of patients with knee OA in clinical practice should include simple foot posture measures, and that the potential influence of foot structure and function on the efficacy of foot orthoses in the management of medial compartment knee OA be further investigated.

## Competing interests

HBM is Editor-in-Chief of the *Journal of Foot and Ankle Research*. It is journal policy that editors are removed from the peer review and editorial decision making processes for papers they have co-authored.

## Authors' contributions

PL: designed and managed the study, collected and analysed the data drafted the manuscript. HBM: participated in the study design and assisted in the statistical analysis and data interpretation, helped to draft the manuscript. RF: assisted in data collection, data analysis. JF, JB and NB have assisted in patient recruitment, grading x-ray severity and drafting the manuscript. PL, HBM and JF obtained the funding. All authors have read and approved the final version.
